# Palliative Healthy Donor Stool Transplantation (pFMT) in Patients with End-Stage Alcohol-Related Cirrhosis and Severe Unstable Decompensations—A Cohort Study

**DOI:** 10.3390/jcm15072607

**Published:** 2026-03-29

**Authors:** Tharun Tom Oommen, Cyriac Abby Philips, Rizwan Ahamed, Arif Hussain Theruvath, Ajit Tharakan, Sasidharan Rajesh, Philip Augustine

**Affiliations:** 1 Clinical and Translational Hepatology, The Liver Institute, Center of Excellence in Gastrointestinal Sciences, Rajagiri Hospital, Aluva 683112, Kerala, India; 2 Department of Clinical Research, The Liver Institute, Center of Excellence in Gastrointestinal Sciences, Rajagiri Hospital, Aluva 683112, Kerala, India; 3 Gastroenterology and Advanced GI Endoscopy, Center of Excellence in Gastrointestinal Sciences, Rajagiri Hospital, Aluva 683112, Kerala, India; 4 Division of Interventional Radiology, Gastroenterology and Hepatology, Center of Excellence in Gastrointestinal Sciences, Rajagiri Hospital, Aluva 683112, Kerala, India

**Keywords:** FMT, ACLF, ALD, sepsis, ascites, acute kidney injury, decompensated cirrhosis

## Abstract

**Background and Aims:** Severe alcohol-associated hepatitis (SAH) can trigger unstable decompensations in cirrhosis patients. They experience high rates of emergency department visits and hospitalization. We evaluated real-world clinical outcomes following palliative-faecal microbiota transplantation (pFMT) compared to best supportive care (BSC) in this critically ill population. **Patients and Methods:** From July 2021 to April 2024, 28 patients on pFMT were compared with 37 on BSC. Patients on pFMT received nasoduodenal healthy donor stool infusion daily for 5-days. Patients were followed up for portal hypertension-related events, infections, hospitalizations, extrahepatic organ failure and 6- and 12-months survival. 16S rRNA sequencing on stool samples collected at baseline and on follow up were analysed for changes in relative abundance (RA) of bacterial communities. **Results:** Patients were matched for age, type of decompensation and liver disease severity at enrolment. Twelve-month survival was 64.3% in pFMT versus 51.4% in BSC groups. pFMT dramatically reduced hospital readmissions (mean 0.76 ± 0.76 vs. 2.29 ± 1.27, *p* < 0.001). Unstable decompensations beyond 3 months occurred in 14.3% of pFMT versus 64.9% of BSC (*p* < 0.001). Organ failures were lesser with pFMT: acute kidney injury 7.7% versus 93.8% (*p* < 0.001), hepatic encephalopathy 7.1% versus 68.2% (*p* < 0.001). Infection burden was significantly lower (53.6% vs. 83.8%, *p* = 0.008), particularly infections requiring admission (17.4% vs. 66.7%, *p* < 0.001) with pFMT. Microbiome analysis revealed progressive expansion of Gram-negative genera in BSC, and beneficial Actinobacteria in pFMT-treated patients at 3, 6, and 12 months. **Conclusions:** Palliative FMT represents a unique disease-modifying intervention in end-stage alcohol-related cirrhosis, preventing organ failure progression, reducing healthcare utilization, and improving survival trajectories.

## 1. Introduction

Patients with severe alcohol-associated hepatitis (AH) superimposed on underlying cirrhosis represent a cohort at the peak of liver disease severity, often presenting with severe unstable decompensations that can progress to acute on chronic liver failure (ACLF) with high short-term mortality [1,2]. This clinical state is characterized by the rapid onset of jaundice and clinical events such as ascites, hepatic encephalopathy, and coagulopathy, driven by intense systemic inflammation and a high susceptibility to infection and organ failure. Additionally, in those with severe AH related ACLF, natural history of the disease is significantly punctuated by clinically relevant (stable or unstable) decompensation events. In the absence of a liver transplant, the prognosis is exceptionally poor, with short-term mortality rates reported to be as high as 50% at three months [3,4]. The current standard of care is limited and primarily involves supportive measures, including absolute alcohol abstinence, intensive nutritional support, and, for those with severe disease (Maddrey’s Discriminant Function score ≥ 32), a course of corticosteroids to suppress inflammation [1,5]. However, a critical treatment gap exists, as approximately 40–50% of these patients are non-responsive to corticosteroid therapy, a status identified by a high Lille score after one week of treatment. Similarly, responders to initial corticosteroid treatment can still present with unstable decompensation beyond 28 days after therapy. For this large subgroup of patients with unstable decompensations, there are no established second-line medical treatments, and mortality can exceed 70% within six months [5]. This leaves these critically ill patients with virtually no effective therapeutic options, other than a curative liver transplantation which, in a developing country like India, without a centralized organ sharing system can sometimes prove challenging [6]. In this context, even the dependence on healthy living related donors may not be a feasible option for many. Without liver transplantation, patients face frequent emergency visits, hospitalizations, and life-threatening events [7]. In low-resource settings, palliative care for unstable decompensated cirrhosis patients focuses on quality of life rather than survival. We aimed to examine the clinical outcomes and overall survival in unstable alcohol-related cirrhosis patients after their index severe AH episode, who received palliative healthy donor stool transplant (pFMT) alongside standard palliation, compared to those given only palliation-based best supportive care (BSC).

## 2. Materials and Methods

### 2.1. Patients

We analysed hospital records to identify patients with probable or definite severe AH from July 2021 to April 2024. The study included those with persistent unstable decompensations two months after their initial presentation, defined as worsening jaundice, new or symptomatic ascites requiring intervention, infections or sepsis needing hospital care, overt hepatic encephalopathy, acute kidney injury, variceal bleeding, or combinations thereof. Exclusion criteria were multiple organ failure needing support, uncontrolled sepsis on inotropes, extrahepatic malignancy, or a strong suspicion of another cause of liver injury. Patients considering liver transplantation were referred accordingly. Written clinical informed consent was obtained from all participants or their immediate family members for pFMT and for the collection and use of anonymized stool samples for future research, as part of standard clinical and research practice. Stool samples were collected at baseline (within 24 h of admission or contact) and at 3, 6, and 12 months in survivors. The study was approved by the Rajagiri Hospital Institutional Review Board and adhered to the Helsinki declaration.

### 2.2. Best Supportive Care Protocol

Patients in the study received best supportive care tailored to liver disease severity. Clinically significant portal hypertension was managed with maximum tolerated beta-blockers, while ascites was treated with the lowest effective dose of diuretics. Hepatic encephalopathy was managed with ongoing rifaximin prophylaxis. Nutritional support included salt restriction and 1–1.5 g/kg/day protein intake. IV albumin was given as indicated during hospitalization. Infections prompted admission and initial empirical therapy with third-generation cephalosporins, adjusted per culture results. Multidisciplinary management addressed infections or acute kidney injury requiring admission, including targeted IV antibiotics, albumin, and terlipressin (or octreotide if needed). Acute variceal bleeding was managed per regional guidelines.

### 2.3. Palliative Healthy Donor Stool Transplant (pFMT) Protocol

**Figure 1:**
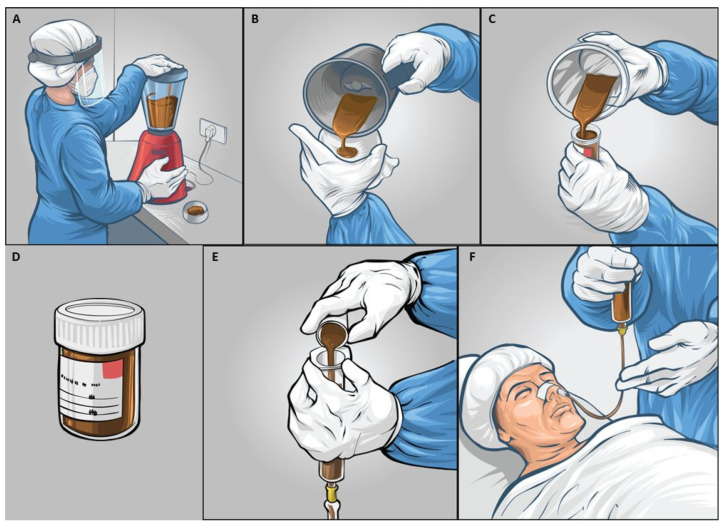
Faecal microbiota transplantation preparation and administration procedure. (**A**) Homogenization of donor stool material using a standard blender under sterile conditions with appropriate personal protective equipment. (**B**) Filtration of faecal suspension through gauze to remove particulate matter. (**C**) Transfer of homogenized faecal suspension from blender container. (**D**) Final filtered faecal suspension in sterile container ready for administration. (**E**) Preparation of faecal suspension in syringe for administration. (**F**) Administration of faecal microbiota transplant to patient via nasoduodenal tube.

For pFMT (Figure 1), fresh donor stool samples (≥30 g) were collected daily and screened according to standard published protocols. Donors provided samples in sterile containers at the hospital within six hours of infusion. Each sample was mixed with 100 mL sterile saline, homogenized using a hand blender (Philips®, Amsterdam, Netherlands) in two-minute timed pulses, and filtered three times through sterile gauze to remove solids. Staff followed safety protocols throughout preparation. The final 150 mL stool suspension was delivered via nasoduodenal tube under fluoroscopic guidance after patients fasted for four hours. Administration occurred daily for seven days, with patients kept supine at a 45-degree angle for 30 min post-infusion, and tubes flushed with 30 mL saline. Disaccharides were allowed to encourage 2–3 soft stools per day, but rifaximin and similar antibiotics were avoided. All other supportive care, except rifaximin, continued during follow-up.

Donors were healthy relatives or unrelated household contacts aged 18–65 years without chronic diseases, recent antibiotic use (within 3 months), or chronic or recent gastrointestinal symptoms. Donor screening included serological testing for hepatitis B and C, HIV, syphilis, and stool examination for ova, cysts, parasites, Clostridium difficile toxin, and multidrug-resistant organisms, as per published global consensus recommendations. Fresh stool was used within 6 h of collection to preserve microbial viability, following protocols established in our prior published experience with FMT in SAH. Nasoduodenal administration was chosen over the rectal route based on prior evidence of superior upper GI engraftment. All preparation was carried out in a designated clean room with staff using appropriate personal protective equipment including N95 masks, sterile gloves, and gowns.

### 2.4. Analysis

#### 2.4.1. General Statistics

Statistical analyses were conducted utilizing the MedCalc Statistical Software v.23.2 (Ostend, Belgium). Data was presented as mean ± standard deviation for normally distributed continuous variables, proportions (%) for categorical variables. Comparisons between groups were performed using chi-square or Fisher’s exact test for categorical variables and Student’s *t*-test or Wilcoxon rank-sum test for continuous variables as appropriate. A *p*-value < 0.05 was considered statistically significant. Survival analysis was performed using Kaplan-Meier method with log-rank test for comparison between groups.

#### 2.4.2. Microbiota Analysis

Stool samples were divided into 500 µg aliquots and stored at −80 °C before DNA extraction. Using the Illumina MiSeq platform, we sequenced the faecal 16S rRNA V3-V4 region from about 200 mg of stool. Bacterial DNA was extracted with an adapted QIAmp DNA Stool Mini Kit protocol. Data analysis was performed using QIIME2 (version 2021.4), which processed raw FASTQ files. The DADA2 algorithm handled quality control by trimming, denoising, and assembling sequences to remove phiX, chimeric, and erroneous reads. Amplicon Sequence Variants (ASVs) were aligned against the GREENGENES database (99% similarity) via QIIME2’s feature-classifier plugin, resulting in a species-level taxonomic table. Relative abundances at baseline and follow-up were displayed as bar.

## 3. Results

### 3.1. Study Population

A total of 65 patients with end-stage alcohol-related cirrhosis and severe unstable decompensations were enrolled, with 28 patients receiving pFMT and 37 receiving BSC. The mean age was 47.8 ± 7.5 years in the pFMT group and 50.4 ± 7.7 years in the BSC group. Diabetes mellitus was the most common comorbidity in both groups (pFMT: 42.9%, BSC: 43.2%). At baseline, both groups had comparable severity scores with mean MELD-3 scores of 30.6 ± 4.7 and 29.6 ± 6.4 for pFMT and BSC groups respectively, and mean Child-Turcotte-Pugh (CTP) scores of 11.2 ± 1.0 and 10.6 ± 1.1 (Table 1).

### 3.2. Unstable Decompensations at Presentation

Progressive/worsening jaundice was present in 60.7% of pFMT patients versus 62.2% of BSC patients. Moderate to severe ascites was observed in 78.6% and 83.8%, overt hepatic encephalopathy in 50.0% and 40.5%, and sepsis in 39.3% and 51.4% of pFMT and BSC groups respectively, all, statistically not significant. The majority of patients required hospitalization at outset (pFMT: 82.1%, BSC: 81.1%) (Table 2).

### 3.3. In-Hospital Outcomes

Death during the index admission occurred in 17.9% of pFMT patients versus 29.7% of BSC patients and mean hospital stay was 14.0 ± 10.0 days for pFMT and 14.1 ± 9.0 days for BSC groups, both of which were statistically not significant.

### 3.4. Post-Treatment Outcomes

**Figure 2:**
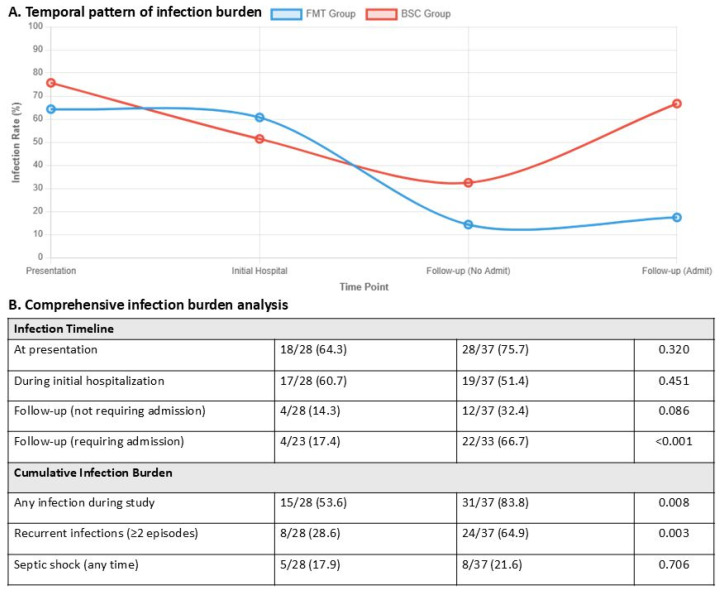
Temporal patterns and comprehensive analysis of infection burden in pFMT versus BSC groups. (**A**) Longitudinal infection rates (%) from presentation through follow-up showing divergent trajectories between pFMT Group (blue line, n = 28) and BSC Group (red line, n = 37) across four time points: presentation, initial hospital admission, follow-up without readmission, and follow-up with readmission. (**B**) Detailed infection burden analysis comparing pFMT Group and BSC Group across infection timeline metrics and cumulative infection burden, with statistically significant differences (*p* < 0.05) highlighted in yellow for any infection during study period (53.6% vs. 83.8%, *p* = 0.008) and recurrent infections ≥ 2 episodes (28.6% vs. 64.9%, *p* = 0.003).

Hospital readmission patterns revealed striking differences between groups, with pFMT patients experiencing significantly fewer readmissions (median 1.0 [IQR 0–1] vs. 2.0 [IQR 1–3]; *p* < 0.001). Notably, only 20% of pFMT patients required multiple readmissions (≥2) compared to 73.5% of BSC patients (χ^2^ = 16.517, *p* < 0.001), while 44% of pFMT patients avoided readmission entirely versus only 2.9% in the BSC group (Table 3). Sepsis/septic shock was the leading cause of readmission in both groups (pFMT: 28.6%, BSC: 37.8%), followed by acute kidney injury (pFMT: 17.9%, BSC: 29.7%) and worsening ascites (pFMT: 17.9%, BSC: 29.7%). The cumulative infection burden was significantly lower in the pFMT cohort (53.6% vs. 83.8%, *p* = 0.008), with marked reduction in infections requiring admission during follow-up (17.4% vs. 66.7%, *p* < 0.001) (Figure 2).

Most remarkably, pFMT demonstrated a profound protective effect against new-onset organ failures, with only 7.7% of at-risk pFMT patients developing new acute kidney injury compared to 93.8% in the BSC group (*p* < 0.001), and similarly, only 7.1% developing new hepatic encephalopathy versus 68.2% in BSC (*p* < 0.001). When examining composite adverse outcomes (defined as death, multiple readmissions, or unstable decompensation), pFMT patients showed significantly better outcomes (50% vs. 89.2%, *p* < 0.001), representing an absolute risk reduction of 39.2% and a number needed to treat of approximately 3. Healthcare resource utilization was dramatically reduced in the pFMT group, with no patients requiring activation of dedicated palliative care services compared to 16.2% in the BSC group (*p* = 0.032), suggesting both improved prognosis and quality of life.

### 3.5. Decompensations Beyond 3 Months

Among patients surviving beyond 3 months, decompensations occurred in 58.3% (14/24) of pFMT patients versus 90.6% (29/32) of BSC patients (*p* < 0.05). Importantly, the pattern of decompensations differed significantly between groups: stable decompensations occurred in 35.7% of pFMT patients versus 24.3% of BSC patients, while unstable decompensations occurred in only 14.3% of pFMT patients compared to 64.9% of BSC patients (χ^2^ = 7.96, *p* < 0.05). Specific decompensations beyond 3 months showed statistically significant differences: persistent/recurrent ascites (pFMT: 43.5% vs. BSC: 75.8%), acute kidney injury (pFMT: 13.0% vs. BSC: 57.6%), persistent/worsening jaundice (pFMT: 21.7% vs. BSC: 78.8%), infections requiring admission (pFMT: 17.4% vs. BSC: 66.7%), encephalopathy (pFMT: 4.0% vs. BSC: 57.6%), and variceal bleeding (pFMT: 7.7% vs. BSC: 48.5%) (Table 4).

### 3.6. Survival Analysis

**Figure 3:**
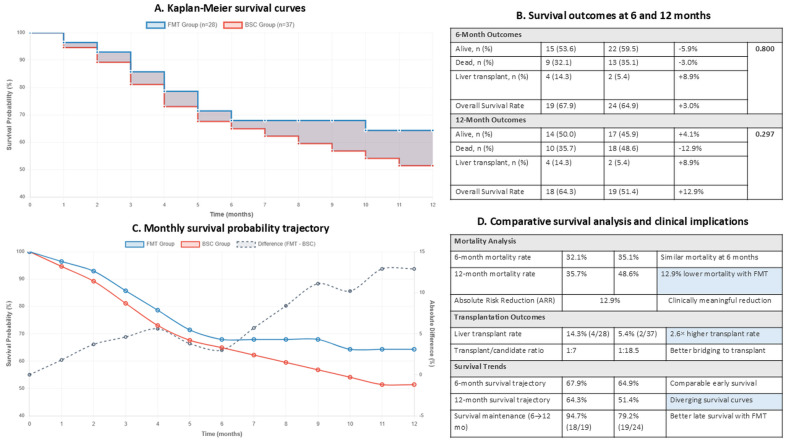
Comparative survival analysis and clinical outcomes between pFMT and BSC groups. (**A**) Kaplan-Meier survival curves comparing pFMT Group (blue, n = 28) versus BSC Group (red, n = 37) over 12 months follow-up. (**B**) Survival outcomes at 6- and 12-months showing mortality rates, liver transplant rates, and overall survival rates with absolute differences and *p*-values. (**C**) Monthly survival probability trajectory for both groups with difference curve (dashed line) demonstrating divergent survival patterns and increasing survival benefit with FMT over time. (**D**) Comparative survival analysis showing key clinical parameters including mortality rates, absolute risk reduction (ARR), number needed to treat (NNT), transplantation outcomes, and survival trajectory patterns, with clinically significant findings highlighted.

Six-month survival rates (including liver transplantation) were 67.9% (19/28) in the pFMT group versus 64.9% (24/37) in the BSC group (*p* ≥ 0.05). At 12 months, survival rates were 64.3% (18/28) in the pFMT group compared to 51.4% (19/37) in the BSC group, representing an absolute difference of 12.9% (*p* = 0.297) which did not reach statistical significance (*p* = 0.297), as our sample size was insufficient to detect differences of this magnitude. Additionally, a higher proportion of pFMT patients underwent liver transplantation (14.3% vs. 5.4%), potentially indicating better clinical stabilization allowing successful bridging to transplant. (Figure 3).

To account for the significantly higher baseline total bilirubin in the pFMT group, multivariable Cox proportional hazards regression was performed. After adjusting for total bilirubin alone, the hazard ratio for 12-month mortality with pFMT was 0.58 (95% CI 0.24–1.37, *p* = 0.21); in the full model adjusting for total bilirubin, age, and MELD-3, the HR was 0.62 (95% CI 0.26–1.44, *p* = 0.26), with MELD-3 as the only independent predictor (HR = 1.10, *p* = 0.01). For the composite adverse outcome (death, multiple readmissions, or unstable decompensation), multivariable logistic regression adjusting for total bilirubin and MELD-3 confirmed that pFMT independently predicted a lower risk (adjusted OR = 0.12, 95% CI 0.03–0.48, *p* = 0.003). These results indicate that the observed benefits of pFMT were not confounded by baseline bilirubin differences.

Sensitivity analyses accounting for differential transplantation rates was performed. When transplant recipients were censored at the time of transplant, 12-month survival was 63.4% in pFMT versus 51.2% in BSC (log-rank *p* = 0.449). When transplant recipients were excluded entirely (pFMT n = 24, BSC n = 35), survival was 57.0% versus 48.4% (log-rank *p* = 0.683). In both analyses, the direction of the survival difference was preserved, confirming that the higher transplant rate in the pFMT group did not inflate the survival estimate.

### 3.7. Microbiota Changes Between Groups

**Figure 4:**
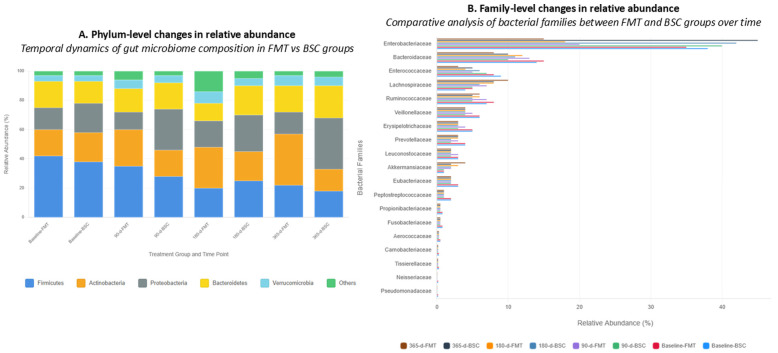
Gut microbiome composition dynamics following pFMT versus BSC treatment. (**A**) Temporal dynamics of gut microbiome composition at phylum level showing relative abundance (%) changes across four time points for both treatment groups, with major phyla including Firmicutes (blue), Actinobacteria (orange), Proteobacteria (grey), Bacteroidetes (yellow), Verrucomicrobia (light blue), and Others (green). (**B**) Family-level changes in relative abundance showing comparative analysis of bacterial families between pFMT and BSC groups at different time points with Enterobacteriaceae showing the most prominent differences between groups.

The longitudinal microbiome analysis reveals profound and divergent trajectories between pFMT and BSC groups over the 12-month follow-up period, demonstrating the disease-modifying impact of gut microbiome restoration. At the phylum level, the most striking finding was the differential evolution of Proteobacteria abundance—a recognized marker of dysbiosis and bacterial translocation in cirrhosis—which remained relatively controlled in the pFMT group while nearly doubling in the BSC group (20% to 35%, representing a 75% relative increase). Conversely, Actinobacteria, which includes beneficial genera with anti-inflammatory properties, showed remarkable expansion in the pFMT group (18% to 35%, a 94% relative increase) while declining in the BSC group (20% to 15%, a 25% relative decrease), suggesting successful engraftment of beneficial commensals. At the family level, Enterobacteriaceae, the predominant pathobiont family associated with endotoxemia and spontaneous bacterial peritonitis in cirrhosis, demonstrated a dramatic 57% relative reduction in the pFMT group (35% to 15%) while increasing by 18% in the BSC group (38% to 45%), correlating with the observed clinical differences in infection rates and organ failure development. The pFMT group also showed progressive increases in beneficial families including Lachnospiraceae (5% to 10%) and maintenance of Ruminococcaceae abundance (8% to 6%), both crucial for short-chain fatty acid production and intestinal barrier integrity, while these families remained suppressed or further declined in the BSC group. Additionally, the emergence of Akkermansiaceae (1% to 4% in pFMT vs. stable at 1% in BSC), known for its mucin-degrading and barrier-protective properties, particularly *Akkermansia muciniphila*, provides mechanistic insight into the improved gut barrier function and reduced bacterial translocation observed clinically (Figure 4).

The overall pattern demonstrates that pFMT successfully interrupted and reversed the dysbiotic trajectory characteristic of unstable decompensated cirrhosis, transitioning from a pathobiont-dominated inflammatory profile to a more balanced state with increased colonization resistance, while the BSC group showed progressive dysbiosis amplification, providing a microbiological foundation for the superior clinical outcomes observed in pFMT-treated patients.

## 4. Discussion

This study represents the first comprehensive real-world evaluation of pFMT as a therapeutic intervention in patients with end-stage alcohol-related cirrhosis presenting with severe unstable decompensations. Our findings demonstrate that pFMT, when added to best supportive care, fundamentally alters the natural history of end-stage cirrhosis. The observed 12-month survival benefit of 12.9% absolute difference (64.3% vs. 51.4%), while not reaching statistical significance in our cohort of 65 patients, represents a clinically meaningful improvement. Notably, the pFMT group had significantly higher baseline total bilirubin reflecting more SAH. Multivariable Cox regression adjusting for this imbalance demonstrated a strengthened effect estimate indicating that the higher baseline bilirubin was biasing the unadjusted estimate against pFMT. Similarly, the composite adverse outcome remained robustly associated with pFMT allocation after multivariable adjustment. This finding of benefit despite greater baseline severity is consistent with previous observations in FMT for SAH, where higher disease severity may be associated with greater dysbiosis and thus plausible greater potential for microbiome restoration [8]. This finding aligns with emerging evidence from recent randomized controlled trials demonstrating survival benefits with gut microbiome modulation in cirrhosis.

Bajaj et al. showed improved cognitive function and reduced hospitalizations with FMT in cirrhotic patients with hepatic encephalopathy [9]. A recent study by Ichim and colleagues showed that FMT demonstrated a favorable safety profile and yielded early clinical and biochemical benefits in patients with cirrhosis [10]. Our study extends these observations to the most critically ill subset, representing a population typically excluded from interventional trials. It suggests that pFMT can alter the natural history of this terminal disease stage, extending life in a population for whom timely liver transplantation is often not an option.

The striking reduction in unstable decompensations beyond 3 months suggests that pFMT may interrupt the progressive spiral of decompensation that characterizes end-stage liver disease. This finding is particularly significant given the concept of “unstable decompensated cirrhosis” recently defined by the Baveno VII consensus, where patients experience a rapidly progressive course with multiple organ failures [11]. The gut-liver axis plays a central role in this progression, with intestinal dysbiosis driving bacterial translocation, systemic inflammation, and subsequent organ failures [12]. By restoring intestinal eubiosis, pFMT appears to break this pathophysiological cascade, as evidenced by the dramatic reduction in new-onset organ failures, particularly the 92% relative risk reduction in new AKI development and 90% reduction in new hepatic encephalopathy.

The profound impact on hospital readmissions, with a 67% reduction in mean readmissions and only 20% of pFMT patients experiencing multiple readmissions compared to 73.5% of BSC patients, has significant implications for healthcare economics and patient quality of life. This finding corroborates recent data showing reduced readmission rates with microbiome-targeted therapies in cirrhosis [13]. However, the magnitude of effect in our severely ill population exceeds previously reported benefits. The parallel reduction in infection burden, particularly infections requiring admission, suggests that pFMT possibly restores colonization resistance and reduces pathobiont dominance, mechanisms that have been elegantly demonstrated in human studies of alcohol-related liver disease [14,15].

A notable finding was the higher liver transplant rate in the pFMT group (14.3% vs. 5.4%), which, despite seeming contradictory to better outcomes, likely indicates effective “bridging”—these patients remained stable enough for evaluation and surgery. Unlike the BSC group, none of the pFMT patients required palliative care, suggesting maintained transplant eligibility. Data from ELITA-CLIF supports the importance of preventing decompensation to avoid delisting [16]. The survival benefit also included fewer clinical events, hospitalizations, sepsis, and hepatic encephalopathy, which are major causes of morbidity and mortality in advanced cirrhosis [17,18].

The results of this study align with and expand upon an emerging body of literature supporting the use of pFMT in severe alcohol-related liver disease. Several pilot studies and early-phase clinical trials have demonstrated the safety and potential efficacy of FMT, particularly in the context of SAH and ACLF [19,20,21,22]. Our study corroborates these findings in a broader, real-world cohort of unstable decompensated cirrhosis, suggesting the benefits of pFMT are not limited to the specific inflammatory milieu of SAH but may apply more generally to the advanced stages of alcohol-related liver disease.

The observed reduction in overt HE is one of the most well-supported outcomes of FMT in cirrhosis. Multiple studies and systematic reviews have concluded that FMT can improve cognitive function and reduce hospitalizations for HE [23]. This effect is believed to be mediated by the restoration of a healthy gut microbiome, which leads to reduced production of ammonia and other neurotoxins, decreased systemic inflammation, and improved intestinal barrier function. The significant reduction in follow-up HE in our pFMT cohort reinforces its role as a potent therapy for this debilitating complication.

The mechanisms underlying pFMT’s beneficial effects likely extend beyond simple microbial replacement. Recent multi-omics studies have identified restoration of bile acid homeostasis, reduced endotoxemia, decreased production of aromatic amino acid metabolites, and enhanced short-chain fatty acid production as key mediators of pFMT efficacy in liver disease and recent works have demonstrated that gut microbiome modulation can improve bile acid profiles and reduce portal pressure in decompensated cirrhosis [12,24,25].

This study uniquely frames pFMT within palliative care for patients with end-stage cirrhosis and unstable decompensations, most of whom are not eligible for transplantation and have limited life expectancy. In this context, care focuses on symptom relief, quality of life, and reducing treatment burden rather than cure. Unlike traditional reactive management that often leads to recurring symptoms and frequent hospitalizations, pFMT targets gut dysbiosis proactively. Our results show pFMT reduces hospitalizations, infections, and HE episodes, directly lessening treatment burden and improving quality of life. This positions pFMT as a foundational palliative strategy, stabilizing disease and minimizing suffering so patients spend more time outside the hospital.

Our findings have several limitations. First, the non-randomized, single-center, retrospective cohort design introduces potential selection bias that cannot be fully mitigated despite comparable baseline demographics, decompensation profiles, and disease severity scores between groups. Unmeasured confounders, including clinician preference, timing of referral, patient willingness to undergo the procedure, and psychosocial factors, may have influenced treatment allocation. Second, the small sample size limits statistical power for detecting modest survival differences and precludes robust subgroup analyses. Third, donor selection followed standard protocols without targeting specific microbial signatures, which may affect reproducibility and efficacy across centers. Finally, we did not collect formal patient-reported outcomes, so direct evidence of palliative benefit on quality of life is inferred rather than measured. Future multi-center randomized controlled trials with adequate sample sizes, standardized FMT protocols, and pre-specified patient-reported outcome measures are essential to validate these findings and establish the role of pFMT in the management of end-stage alcohol-related cirrhosis.

Regarding the pFMT protocol, several limitations warrant discussion. The use of fresh donor stool rather than standardized frozen preparations introduces batch-to-batch variability and limits scalability. Additionally, while our donor screening was comprehensive, it was based on contemporaneous guidelines and did not include metagenomic screening for antibiotic resistance genes or viral metagenomics. The 5–7-day treatment course was empirically derived from ours and others prior published experience. Despite these limitations, pFMT-related immediate adverse events occurred in 25% of patients, comprising fever and worsening sepsis—events that are difficult to distinguish from the natural disease course in this critically ill population. Delayed events unrelated to liver disease included dermatological manifestations and metabolic decompensation. No life-threatening adverse events were directly attributable to pFMT.

Our study framed pFMT within a palliative intent for patients with end-stage cirrhosis and unstable decompensations with limited life expectancy. While we did not employ formal patient-reported outcome measures or validated quality-of-life instruments, the objective clinical surrogates—including significantly fewer hospitalizations, elimination of the need for dedicated palliative care services, and reduced organ failure burden—provide indirect but compelling evidence that pFMT reduces suffering and treatment burden in this population. Future studies should incorporate validated instruments such as the Chronic Liver Disease Questionnaire (CLDQ) or EQ-5D to directly measure the impact on patient-perceived quality of life.

Future research should focus on several key areas: (1) identification of optimal donor characteristics and microbial signatures associated with therapeutic success; (2) determination of optimal timing, route, and frequency of pFMT administration; (3) evaluation of pFMT in combination with other microbiome-targeted interventions such as precision probiotics or bacteriophage therapy; (4) assessment of pFMT’s impact on portal hypertension and hepatic hemodynamics; and (5) long-term outcomes including hepatocellular carcinoma risk and post-transplant survival. Additionally, mechanistic studies employing multi-omics approaches are needed to understand how pFMT interrupts the pathophysiological cascade of decompensated cirrhosis.

## 5. Conclusions

In summary, this single-center cohort study provides preliminary evidence that pFMT, when added to best supportive care, is associated with significantly fewer hospital readmissions, reduced infection burden, markedly lower rates of new-onset organ failures (acute kidney injury and hepatic encephalopathy), and fewer unstable decompensations in patients with end-stage alcohol-related cirrhosis. While a 12.9% absolute difference in 12-month survival favouring pFMT was observed, this did not reach statistical significance in our sample of 65 patients, and confirmation in adequately powered studies is warranted. The microbiome data suggests a plausible biological mechanism through reversal of dysbiosis. These findings support pFMT as a promising adjunctive intervention in this critically ill population and provide the rationale and effect-size estimates needed for the design of future randomized controlled trials.

## Figures and Tables

**Table 1:** Baseline demographic, clinical characteristics and laboratory parameters.

Characteristic	FMT Group (n = 28)	BSC Group (n = 37)	*p*-Value
*Demographics*			
Age (years), mean ± SD	47.8 ± 7.5	50.4 ± 7.7	0.172
Male sex, n (%)	28 (100)	37 (100)	1.000
*Comorbidities, n (%)*			
Diabetes mellitus	12 (42.9)	16 (43.2)	0.976
Hypertension	4 (14.3)	2 (5.4)	0.230
Obesity/Overweight	4 (14.3)	1 (2.7)	0.084
Dyslipidaemia	2 (7.1)	3 (8.1)	0.885
Chronic kidney disease	1 (3.6)	0 (0)	0.431
HCC at presentation	1 (3.6)	2 (5.4)	0.728
*Severity Scores, mean ± SD*			
MELD-3	30.6 ± 4.7	29.6 ± 6.4	0.479
Child-Turcotte-Pugh	11.2 ± 1.0	10.6 ± 1.1	0.053
ALBI score	−0.71 ± 0.29	−0.82 ± 0.46	0.271
Maddrey’s Discriminant Function	89.3 ± 39.9	82.1 ± 63.0	0.595
Haemoglobin (g/dL)	10.5 ± 1.8	9.8 ± 2.1	0.152
Total leucocyte count (×10^3^/µL)	11.8 ± 7.5	10.1 ± 5.6	0.318
Platelet count (×10^3^/µL)	130.3 ± 56.0	113.7 ± 36.5	0.163
Total bilirubin (mg/dL)	16.7 (11.3–23.3)	7.4 (3.5–12.8)	<0.001
AST (IU/L)	162.0 ± 103.4	136.0 ± 91.5	0.282
ALT (IU/L)	73.1 ± 43.6	64.7 ± 69.4	0.572
Alkaline phosphatase (IU/L)	178.0 ± 81.8	180.9 ± 108.5	0.906
Serum albumin (g/dL)	2.5 ± 0.3	2.5 ± 0.6	0.914
Blood urea (mg/dL)	39.3 ± 37.5	45.1 ± 37.7	0.534
Serum creatinine (mg/dL)	1.3 ± 1.2	1.5 ± 1.0	0.428
Serum sodium (mEq/L)	128.8 ± 5.9	130.9 ± 7.0	0.189
INR	2.5 ± 0.8	2.7 ± 2.1	0.571

*Abbreviations: AST, aspartate aminotransferase; ALT, alanine aminotransferase; INR, international normalized ratio. Data presented as mean ± standard deviation or as median (IQR).*

**Table 2:** Unstable decompensations prior to treatment.

Decompensation Type	FMT Group, n (%)	BSC Group, n (%)	*p*-Value
Progressive/worsening jaundice	17 (60.7)	23 (62.2)	0.905
Persistent jaundice	11 (39.3)	14 (37.8)	0.905
Moderate to severe ascites	22 (78.6)	31 (83.8)	0.594
Overt hepatic encephalopathy	14 (50.0)	15 (40.5)	0.449
Sepsis	11 (39.3)	19 (51.4)	0.334
Acute kidney injury	15 (53.6)	21 (56.8)	0.798
Recurrent sepsis	5 (17.9)	3 (8.1)	0.235
Acute variceal bleeding	4 (14.3)	11 (29.7)	0.143
Hospitalization at outset	23 (82.1)	30 (81.1)	0.913
Septic shock	5 (17.9)	8 (21.6)	0.706
Infection at outset	18 (64.3)	28 (75.7)	0.320

**Table 3:** Hospital readmissions post treatment between groups.

Parameter	FMT Group (n = 25) *	BSC Group (n = 34) *	*p*-Value
*Readmission Statistics*			
Mean ± SD	0.76 ± 0.76	2.29 ± 1.27	<0.001 †
Median (IQR)	1.0 (0.0–1.0)	2.0 (1.0–3.0)	
Range	0–2	0–6	
*Distribution of Readmissions, n (%)*			
0 admissions	11 (44.0)	1 (2.9)	<0.001 ‡
1 admission	9 (36.0)	8 (23.5)	
2 admissions	5 (20.0)	14 (41.2)	
3 admissions	0 (0)	5 (14.7)	
4 admissions	0 (0)	4 (11.8)	
≥5 admissions	0 (0)	2 (5.9)	
*Readmission Categories*			
No readmission	11 (44.0)	1 (2.9)	<0.001 §
Single readmission	9 (36.0)	8 (23.5)	
Multiple readmissions (≥2)	5 (20.0)	25 (73.5)	

* Excludes patients who died during index admission; † Mann-Whitney U test (U = 130.5, z = 4.517); Independent t-test (t = 5.760); ‡ Chi-square test for distribution; § Chi-square test for multiple readmissions (χ^2^ = 16.517).

**Table 4:** Decompensation beyond 3 months and organ failure development and progression.

Decompensations Beyond 3 Months		pFMT	BSC	*p*-Value
Any decompensation, n/N (%)		14/24 (58.3)	29/32 (90.6)	0.004
Stable decompensation, n (%)		10/28 (35.7)	9/37 (24.3)	0.318
Unstable decompensation, n (%)		4/28 (14.3)	24/37 (64.9)	<0.001
**Organ system**	**Time point**	**pFMT, n/N (%)**	**BSC, n/N (%)**	***p*** **-value**
Kidney	Baseline AKI	15/28 (53.6)	21/37 (56.8)	0.798
	Follow-up AKI/AKD	3/23 (13.0)	19/33 (57.6)	<0.001
	New-onset AKI *	1/13 (7.7)	15/16 (93.8)	<0.001
Brain	Baseline overt HE	14/28 (50.0)	15/37 (40.5)	0.449
	Follow-up HE	1/25 (4.0)	19/33 (57.6)	<0.001
	New onset HE *	1/14 (7.1)	15/22 (68.2)	<0.001
Portal hypertension	Follow-up variceal bleed	2/26 (7.7)	16/33 (48.5)	<0.001

* New onset = developed during follow-up in patients without this complication at baseline; AKD = Acute Kidney Disease; HE = Hepatic encephalopathy.

## Data Availability

The datasets generated during this study are not publicly available due to ethical restrictions and patient privacy considerations, as the institutional ethics approval did not permit open data deposition. Anonymized data supporting the findings are available from the corresponding author upon reasonable request, subject to institutional data sharing agreements.

## Abbreviations

**ACLF**	acute on chronic liver failure
**AH**	alcohol-associated hepatitis
**AKD**	Acute Kidney Disease
**BSC**	best supportive care
**CTP**	Child-Turcotte-Pugh
**HE**	Hepatic encephalopathy
**pFMT**	palliative faecal microbiota transplantation
**RA**	relative abundance
**SAH**	Severe alcohol-associated hepatitis
